# A New Approach for Achieving Earlier and More Accurate Diagnosis of Connective Tissue Disease-Related Interstitial Lung Disease: *TGFB* and *PDGFA* as Novel Promising Biomarkers

**DOI:** 10.3390/ijms262110722

**Published:** 2025-11-04

**Authors:** Verónica Pulito-Cueto, Belén Atienza-Mateo, Joao C. Batista-Liz, Rebeca Nieto-Nieto, Clara Vaquera-Illescas, María Sebastián Mora-Gil, David Iturbe-Fernández, Víctor M. Mora-Cuesta, Ana Serrano-Combarro, Sheila Izquierdo-Cuervo, Carolina Aguirre Portilla, José M. Cifrián, Ricardo Blanco, Raquel López-Mejías

**Affiliations:** 1 Immunopathology Group, Marqués de Valdecilla University Hospital-IDIVAL, 39011 Santander, Spainjoabatis1995@gmail.com (J.C.B.-L.); rnieton15@gmail.com (R.N.-N.); claravaqueraille@hotmail.com (C.V.-I.); mariasebastianmora@gmail.com (M.S.M.-G.); diturfer@gmail.com (D.I.-F.); anaserco@hotmail.com (A.S.-C.); sheila.izquierdo@scsalud.es (S.I.-C.); carolinaaguirreportilla@gmail.com (C.A.P.); raquel.lopez@idival.org (R.L.-M.); 2 Department of Rheumatology, Hospital Universitario Marqués de Valdecilla, 39008 Santander, Spain; 3 Department of Pneumology, Hospital Universitario Marqués de Valdecilla, 39008 Santander, Spain

**Keywords:** interstitial lung disease, connective tissue diseases, rheumatoid arthritis, systemic sclerosis, inflammatory myopathies, biomarkers, pulmonary fibrosis, profibrotic genes

## Abstract

An early and accurate diagnosis of connective tissue diseases-related interstitial lung disease (CTD-ILD) is crucial for delaying lung fibrosis, but its unknown etiology and the limitations of clinical tools make it challenging for clinicians. *PDGF* and *TGFB* are the main profibrotic genes. We evaluated *PDGFA*, *TGFB1*, *TGFB2*, and *TGFB3* role in the diagnosis of ILD associated with rheumatoid arthritis (RA), systemic sclerosis (SSc), and inflammatory myopathies (IM). Blood was collected from 289 patients:33 RA-ILD, 31 SSc-ILD, 29 IM-ILD; and 22 RA-nonILD, 18 SSc-nonILD, 8 IM-nonILD; and 148 idiopathic pulmonary fibrosis (IPF). The relative expression was quantified by qPCR. Lower *PDGFA*, *TGFB1*, and *TGFB2* expression differentiated RA-ILD from RA-nonILD patients, acting as ILD early diagnostic biomarkers in RA with cut-offs of <0.01153, <0.3185, and <0.001410, respectively. SSc-ILD patients revealed decreased *TGFB2* expression compared to SSc-nonILD patients, with a cut-off of <0.0018 identifying ILD in SSc. *PDGFA* and *TGFB2* expression discriminated IM-ILD from IPF acting as accurate diagnostic biomarkers with cut-offs of >0.0166 and >0.001547, respectively. *PDGFA* and *TGFB2*, as well as *TGFB2* and *TGFB3* expression were associated with RA-ILD and SSc-ILD prognosis, respectively. *PDGFA* and *TGFB* are promising blood biomarkers with clinical value for the early and accurate CTD-ILD diagnosis.

## 1. Introduction

Myofibroblasts are the cellular denominator common to all forms of fibrosis studied and have been postulated as the determining cell in active and irreversible fibrosis development [1,2,3,4,5]. There are several hypotheses about the origin of myofibroblasts, although the best-studied mechanism is transdifferentiation from fibroblasts. Following lung injury, quiescent fibroblasts are activated and transformed into myofibroblasts resulting in excessive accumulation of cellular matrix components [2,3,4,6]. Interestingly, transforming growth factor-β (TGF-β) is considered the main profibrogenic agent as its actions are predominantly attributed to its critical role in the phenoconversion of fibroblasts to myofibroblasts and the protection it provides to these cells against cell death by apoptosis, perpetuating the fibrogenic process [1,2,3,4,5]. In this sense, TGF-ligands consisting of TGFβ-1, TGFβ-2, and TGFβ-3 are known to participate in various cellular processes, including differentiation, proliferation, migration, extracellular matrix remodeling, and apoptosis, all of which influence embryogenesis, wound healing, fibrosis, inflammation, and tumor progression [2]. Importantly, other factors involved in myofibroblast differentiation have been described, of which platelet-derived growth factor (PDGF) is one of the most relevant. Thus, PDGF plays an essential role in the biological processes of homeostasis and tissue repair, especially in the lung. Following tissue injury, PDGF receptor expression is induced in fibroblasts during the fibrotic process, leading to fibroblast proliferation and stimulating fibroblast extracellular matrix synthesis and the release of profibrotic mediators, contributing to myofibroblast formation to facilitate tissue repair [7]. In the last decade, PDGF has been better characterized, and it is now known as a family of four cystine-knot-type growth factors, being PDGF-AA, which binds specifically to PDGF receptor αα, which signaling controls the development of several organs such as lungs [7].

Interestingly, it has been described that myofibroblast foci adjacent to lesions in the damaged lung epithelium are responsible for the abnormal and increased deposition of alveolar and intra-alveolar extracellular matrix, as occurs in the airway of fibrosing processes [2,3,4,6]. In this context, lung fibrosis development constitutes one of the most potentially fatal complications in patients with connective tissue disease (CTD), interstitial lung disease (ILD) being the most common. Indeed, ILD is the leading cause of mortality in patients with CTD, mainly in those with systemic sclerosis (SSc), rheumatoid arthritis (RA), and inflammatory myopathies (IM), among others [8,9,10], being crucial its early diagnosis. Unfortunately, there are still multiple difficulties in diagnosing CTD-ILD, which is a matter of great concern. Firstly, patients may be asymptomatic until the late stages of the disease or present with non-specific symptoms [11], and there is a lack of clearly established protocols for identifying them. Secondly, current assessment methods, including evaluating signs and symptoms, performing pulmonary function tests (PFTs), and using high-resolution computed tomography (HRCT), and, in special cases, lung tissue biopsy, are limited in their ability to diagnose CTD-ILD patients accurately and early on. PFTs are very useful for monitoring the progression of ILD, but they are not specific enough to be used as diagnostic tools since lung function declines slowly and is not easy to detect [12]. In fact, patients in earlier phases of ILD may be asymptomatic and still have normal lung function [12]. Thus, HRCT is the gold standard in the diagnosis of pulmonary fibrosis, but it has a certain delay, there is a low to moderate agreement among expert radiologists interpreting it, and it is associated with radiation harmful to humans, which means it requires careful indication [12]. Although lung tissue biopsy is used in special cases of ILD, it is associated with significant limitations, including its invasive nature, the risk of complications, limited feasibility in patients with advanced disease or comorbidities, and increased morbidity and mortality following its use [13]. Consequently, the early diagnosis of ILD in patients with CTD often remains a challenge for clinicians and, given its poor prognosis and emerging immunomodulatory and antifibrotic treatment options [11], there is considerable interest in addressing this problem. Finally, the difficulty of accurately identifying CTD-ILD is also affected by the fact that it shares pathological features with other lung conditions that have different prognoses and require different therapies, such as idiopathic pulmonary fibrosis (IPF) [14]. Consequently, delayed identification of CTD-ILD can result in patients progressing to more severe stages of lung pathology, with irreversible damage evident at the time of diagnosis. In this sense, detecting early molecular changes before abnormalities appear in imaging or functional tests could facilitate diagnosis. Therefore, circulating biomarker levels represent attractive tools as an accessible and less invasive diagnostic method for complementing the limitations of current methods and guiding clinicians in diagnosing CTD-ILD in cases of clinical uncertainty, where the link between pulmonary fibrosis and CTD is unclear, and the symptoms are atypical and not obvious. Our group and others have already addressed this field in the context of ILDs [15,16], although CTD-ILD has been little explored [5,17,18,19,20,21,22]. Interestingly, TGFβ and PDGFA play a key role in the development of all the studied fibroses, and their profibrotic activity has been observed in lung-resident cells, including fibroblasts, myofibroblasts, endothelial cells, and alternatively activated macrophages [1,2,3,4,5,6,7]. Peripheral blood cells could provide a surrogate source of biomarkers to complement the current methods for diagnosing CTD-ILD in a minimally invasive and more accessible manner. Nevertheless, the expression of *TGFB* and *PDGFA* in the bloodstream has not yet been investigated as potential markers in the pathogenesis of CTD-ILD.

Accordingly, the aim of this study was to evaluate the role of *PDGFA*, *TGFB1*, *TGFB2*, and *TGFB3* as circulating blood biomarkers with clinical value in the early and accurate diagnosis of CTD-ILD.

## 2. Results

### 2.1. Alterations of the PDGFA, TGFB1, and TGFB2 Expression in the Blood Are Associated with the Presence of ILD in RA and SSc Patients

**Figure 1:**
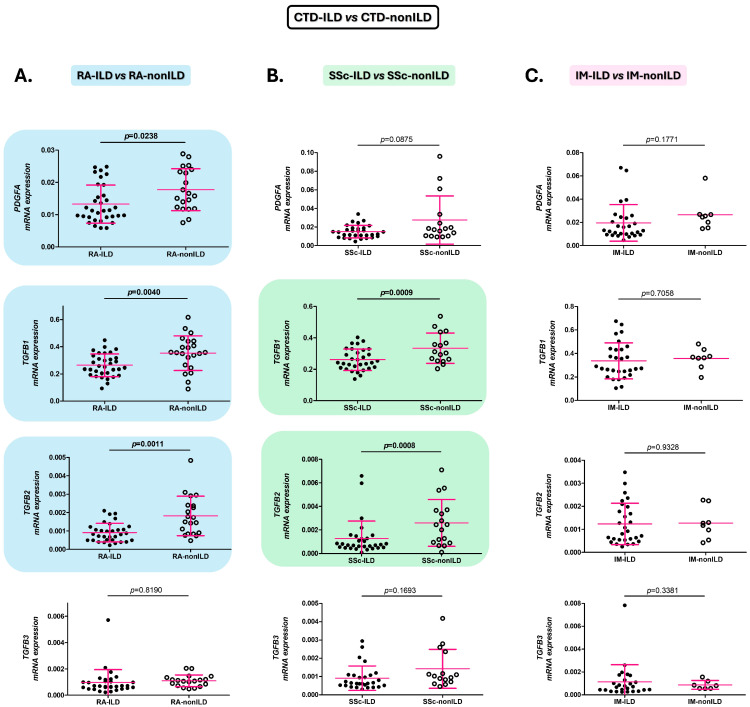
Role of *PDGFA*, *TGFB1*, *TGFB2*, and *TGFB3* in the identification of ILD in CTD patients. Differences in *PDGFA*, *TGFB1*, *TGFB2*, and *TGFB3* expression in blood of patients with RA-ILD versus those with RA-nonILD (**A**), with SSc-ILD versus those with SSc-nonILD (**B**), and with IM-ILD versus those with IM-nonILD (**C**). CTD: connective tissue diseases; ILD: interstitial lung disease; RA: rheumatoid arthritis; *PDGFA*: platelet-derived growth factor subunit A; *TGFB*: transforming growth factor beta; SSc: systemic sclerosis; IM: inflammatory myopathy. Significant results are highlighted.

A statistically significant decrease in expression of *TGFB1* and *TGFB2* was observed in patients with RA-ILD and SSc-ILD compared to those with RA-nonILD and SSc-nonILD, respectively (*TGFB1*: *p* = 0.0040, Figure 1A and *p* = 0.0009, Figure 1B, respectively; and *TGFB2*: *p* = 0.0011, Figure 1A and *p* = 0.0008, Figure 1B, respectively).

In addition, patients with RA-ILD exhibited significantly lower *PDGFA* expression than those with RA-nonILD (*p* = 0.0238, Figure 1A).

However, patients with IM-ILD and IM-nonILD presented similar levels of *PDGFA*, *TGFB1*, and *TGFB2* expression (Figure 1C).

Likewise, *TGFB3* gene expression did not reveal significant differences between either group of patients with CTD-ILD and those with CTD-nonILD (Figure 1A–C).

### 2.2. Alterations of the PDGFA and TGFB2 Expression in the Blood Distinguish Patients with IM-ILD from Those with IPF

**Figure 2:**
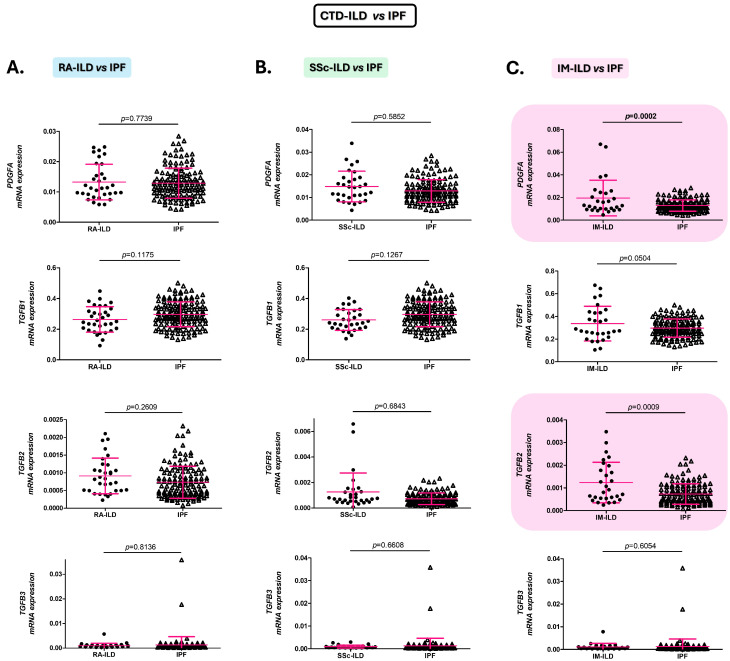
Role of *PDGFA*, *TGFB1*, *TGFB2*, and *TGFB3* in the discrimination between patients with CTD-ILD and IPF. Differences in *PDGFA*, *TGFB1*, *TGFB2*, and *TGFB3* expression in blood of patients with RA-ILD versus those with IPF (**A**), with SSc-ILD versus IPF patients (**B**), and with IM-ILD versus those with IPF (**C**). CTD: connective tissue diseases; ILD: interstitial lung disease; IPF: idiopathic pulmonary fibrosis; RA: rheumatoid arthritis; *PDGFA*: platelet-derived growth factor subunit A; *TGFB*: transforming growth factor beta; SSc: systemic sclerosis; IM: inflammatory myopathy. Significant results are highlighted.

IM-ILD patients presented statistically significant increases in *PDGFA* and *TGFB2* expression compared to IPF patients (*p* = 0.0002 and *p* = 0.0009, respectively, Figure 2C). No statistically significant differences were observed in *TGFB1* and *TGFB3* gene expression between the two groups (Figure 2C).

Similarly, patients with RA-ILD and SSc-ILD revealed similar expression of the analyzed genes (*PDGFA*, *TGFB1*, *TGFB2*, and *TGFB3*) to IPF patients (Figure 2A,B).

### 2.3. PDGFA, TGFB1, and TGFB2 as Early Diagnostic Biomarkers of the Presence of ILD in RA and SSc

**Figure 3:**
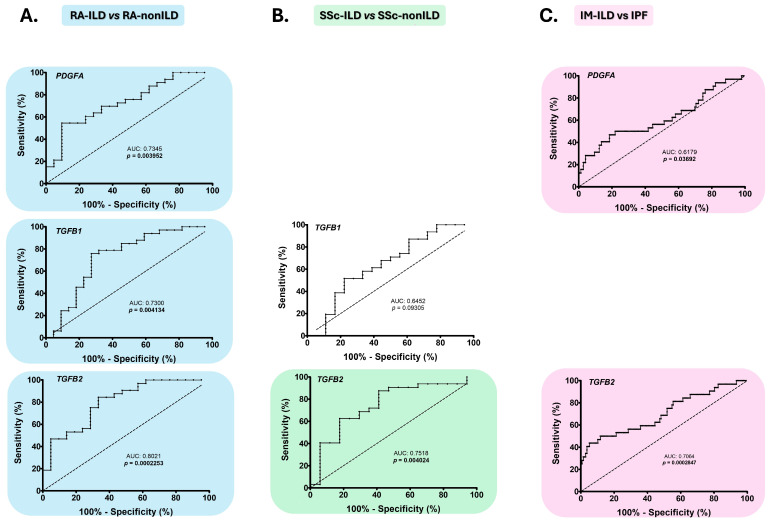
Role of *PDGFA*, *TGFB1*, and *TGFB2* as biomarkers for early diagnosis of ILD in CTD, and for differential diagnosis between CTD-ILD and IPF. ROC curve analysis of *PDGFA*, *TGFB1*, and *TGFB2* to discriminate patients with RA-ILD and RA-nonILD (**A**), SSc-ILD and SSc-nonILD (**B**) and IM-ILD and IPF (**C**). CTD: connective tissue diseases; RA: rheumatoid arthritis; ILD: interstitial lung disease; PDGFA: platelet-derived growth factor subunit A; AUC: area under the curve; TGFB: transforming growth factor beta; SSc: systemic sclerosis; IM: inflammatory myopathy; IPF: idiopathic pulmonary fibrosis. Significant results are highlighted.

ROC curve analysis confirmed that the assessment of *PDGFA*, *TGFB1*, and *TGFB2* expression effectively differentiated between RA-ILD and RA-nonILD patients, acting as biomarkers for the early detection of ILD in RA (AUC: 0.7345, *p* = 0.0040, AUC: 0.7300, *p* = 0.0041, AUC: 0.8021, *p* = 0.0002, respectively, Figure 3A, Table S1). The optimal cut-off values that achieved the best sensitivity and specificity for *PDGFA*, *TGFB1*, and *TGFB2* to identify ILD in RA were <0.01153, <0.3185, and <0.001410, respectively (Table S1).

Furthermore, *TGFB2* expression demonstrated the ability to discriminate between patients with SSc-ILD and those with SSc-nonILD, acting as an early diagnostic biomarker of ILD in SSc (AUC: 0.7518, *p* = 0.0040, Figure 3B, Table S1). The optimal cut-off value for the highest sensitivity and specificity was <0.0018 for *TGFB2* (Table S1). Although statistically significant differences in *TGFB1* gene expression were found in patients with SSc-ILD compared to those with SSc-nonILD, ROC analysis did not confirm that *TGFB1* had sufficient capacity to differentiate between these two groups of patients (Figure 3B, Table S1).

### 2.4. PDGFA and TGFB2 as Biomarkers for the Differential Diagnosis of IM-ILD and IPF Patients

ROC analysis revealed that differences in *PDGFA* and *TGFB2* gene expression were sufficient to differentiate between IM-ILD and IPF patients, indicating that they act as accurate diagnostic biomarkers for IM-ILD (AUC: 0.6179, *p* = 0.0369 and 0.7064, *p* = 0.0003, respectively; Figure 3C, Table S1). The optimal cut-off values for diagnosing IM-ILD vs. IPF with the best sensitivity and specificity were >0.0166 for *PDGFA* and >0.001547 for *TGFB2* (Table S1).

### 2.5. Changes in PDGFA, TGFB2, and TGFB3 Expression Are Associated with the Prognosis of RA-ILD and SSc-ILD Patients

A correlation was observed between *PDGFA* and the radiological pattern on HRCT in patients with RA-ILD, as evidenced by significantly lower gene expression in patients with usual interstitial pneumonia (UIP), probably UIP or indeterminate for UIP pattern than in those with a radiological pattern of non-specific interstitial pneumonia (NSIP) or non-NSIP (0. 0120 ± 0.0058 vs. 0.0161 ± 0.0053, respectively, *p* = 0.044, Table 1). A significant decrease in *TGFB2* expression was also found in patients positive for anti-cyclic citrullinated peptide (anti-CCP) antibodies compared to their counterparts (0.0009 ± 0.0005 vs. 0.0032 ± 0.0048, *p* = 0.010, Table 1). No significant differences were observed in the expression of the analyzed genes in relation to other clinical characteristics, such as the presence of progressive fibrosing pathology, the use of different treatments, RA and ILD duration, and PFRs (Table 1).

For SSc-ILD patients, we observed a positive correlation between *TGFB2* gene expression and FEV1 (% predicted), the lower the gene expression, the lower the FEV1 (r = 0.40, *p* = 0.04, Table 2). We also found a correlation between *TGFB3* expression and the radiological pattern on HRCT, showing elevated *TGFB3* expression with the presence of a UIP pattern, a probably UIP or an indeterminate for UIP compared with a NINE or a non-UIP pattern (0.0069 ± 0.0177 vs. 0.0010 ± 0.0007, *p* = 0.021, Table 2). Additionally, the development of progressive fibrosing pathology influenced *TGFB3* expression, which was higher in SSc-ILD patients with this complication (0.0073 ± 0.0176 vs. 0.0009 ± 0.0006, *p* = 0.024, Table 2). Regarding the clinical characteristics of antibody presence, treatment use, SSc and ILD duration, and PFRs, the expression of the studied genes was similar (Table 2).

Regarding patients with IM-ILD, no significant differences were found in the expression of *PDGFA*, *TGFB1*, *TGFB2*, and *TGFB3* for any of the clinical characteristics evaluated (Table 3).

## 3. Discussion

An ILD diagnosis poses a real challenge and is often only made by excluding other diseases. This increases the likelihood that patients will only be identified at a late stage of the disease, when therapeutics have limited benefit [8,9,10,11,14]. Currently, diagnostic tools available have significant limitations, and there are no clinically useful biomarkers for CTD-ILD. Thus, this work focuses on evaluating the role of *PDGFA*, *TGFB1*, *TGFB2*, and *TGFB3* as possible circulating blood biomarkers for the early and accurate diagnosis of CTD-ILD.

Our results demonstrate, for the first time, that evaluating the expression of the profibrotic factors *PDGFA* and *TGFB* could be key to developing complementary tools that would help overcome the difficulty of diagnosing CTD-ILD.

Early identification of ILD in RA, SSc, and IM is crucial due to the high morbidity and mortality associated with the development of lung disease [8,11,23,24]. To address this issue, our study revealed that the blood gene expression of *PDGFA*, *TGFB1*, and *TGFB2* can distinguish between patients with RA-ILD and those with RA-nonILD, with expression being lower in those patients with ILD. The role of TGF-β protein in fibrosis is well-established [25,26,27], being identified as a biomarker to characterize the disease [28]. Likewise, other studies have shown that the TGF-β1, as well as PDGF-AB (an isoform of PDGF), are present in higher concentrations in the serum of RA-ILD patients compared to those without ILD [29,30]. It is well-known that the biological processes of transcription and translation are susceptible to numerous regulatory mechanisms that adapt gene activity to the organism’s needs. Therefore, it is logical to think that increased levels of the protein form activate a negative feedback process that suppresses gene expression in the blood, indicating a compensatory mechanism. In line with our hypothesis, other authors have found higher PDGF-AB protein levels in the bronchoalveolar lavage of RA-ILD patients compared to those with RA-nonILD [31], again supporting the proposed compensatory mechanism. It is noteworthy that *TGFB2* exhibited similar behavior in patients with SSc-ILD, as its blood gene expression was able to differentiate patients with SSc with and without ILD, with lower expression observed in patients with ILD. The TGF-β contribution in the pathophysiology of SSc and its clinical manifestations, such as lung involvement, have previously been demonstrated [32,33,34]. In addition, several studies have suggested that TGF-β could be a potential biomarker for SSc-ILD, as it is present in increased amounts in fibrotic tissues [35,36]. Taken together, these studies and our results reinforce the previously proposed idea that the lower *TGFB2* blood expression of SSc-ILD patients is the consequence of a compensatory regulatory mechanism that represses gene expression because of overexpression at serum and tissue levels.

Due to the similarity between CTD-ILD and IPF in terms of clinical, radiological, and pathological features, it is essential to distinguish between them correctly [14]. Our study revealed that *PDGFA* and *TGFB2* discriminate IM-ILD from IPF, with higher gene expression observed in patients with the underlying CTD. Consistent with our results, other authors showed a lower *PDGFA* expression in IPF patients vs. healthy subjects [37], decreased serum levels of PDGF-AA in patients with IM vs. healthy subjects [38] and explained the important role of TGF-β in both IPF [39,40,41] and IM [42,43]. However, our research is the first to reveal that *PDGFA* and *TGFB2* could act as biomarkers to distinguish this disease from IPF. Therefore, we can see that patients with pulmonary fibrosis, whether CTD-ILD or IPF, are characterized by lower gene expression of the studied profibrotic factors in blood than patients without such fibrosis, such as those with CTD-nonILD. We propose again that the overexpression of the protein at the tissue and serum levels may trigger a compensatory mechanism that regulates blood gene expression. Furthermore, in cases of more severe pulmonary fibrosis, such as IPF, there is a more pronounced decrease in expression, possibly in response to higher levels of protein production in the tissues and serum than in patients with pulmonary fibrosis and an inflammatory component, who have a better prognosis, such as those with CTD-ILD.

It is noteworthy that the overlap in biomarker levels observed in some cases of CTD-ILD, CTD-nonILD, and IPF patients could be reflecting the biological heterogeneity and complexity of ILD in CTD, where inflammatory and fibrotic processes coexist to varying degrees. Interestingly, the ROC analysis confirmed the discriminative ability of most CTD-ILD biomarkers to be moderate, with AUC values ranging from 0.7 to 0.8 (commonly interpreted as “acceptable” discriminative performance in biomedical research [44]), and a satisfactory performance (AUC = 0.802) for *TFGB2* in differentiating RA-ILD from RA-nonILD. Accordingly, and interpreting the values in the context of biological variability and limited sample size, our results suggest that these biomarkers could be useful in a clinical context for diagnosing CTD-ILD as part of a broader panel and combined with other clinical, imaging, or functional parameters.

It is important to identify the severity of CTD-ILD to halt the progression of fibrosis before irreversible damage occurs. Notably, our results proposed *PDGFA* as a prognostic indicator of RA-ILD severity, as its gene expression were lower in patients with radiological patterns considered to be more severe with a worse prognosis (UIP, probably UIP or an indeterminate radiological pattern) than in patients with better prognosis patterns (NINE or non-NINE) [8]. Furthermore, we also found lower *TGFB2* expression in RA-ILD patients who had circulating anti-CCP antibodies, which are associated with a higher incidence of ILD in RA and its progression to more severe forms [45]. No previous research has investigated these correlations with *PDGFA* and *TGFB2*, being the first study to reveal their important role in RA-ILD prognosis. These findings are consistent with our hypothesis, since low levels of gene expression are once again associated with the presence of clinical features typical of pulmonary fibrosis development and a worse prognosis.

Additionally, in patients with SSc-ILD we found a positive correlation between *TGFB2* expression levels and FEV1 (% predicted), indicating that lower *TGFB2* expression is associated with poorer lung function. A previous study also found a significant correlation between sputum TGF-β molecule levels and annual FEV1, concluding that higher concentrations of this protein are associated with greater lung function deterioration over time [46]. This study reinforced our hypothesis, since elevated TGF-β levels, together with lower *TGFB2* gene expression, as a compensatory mechanism, are related to worse lung function. Moreover, *TGFB3* gene expression was higher in those with the most severe radiological pattern (NIU pattern) and progressive fibrosing pathology. Although *TGFB3* has been detected in fibrotic lung tissue, it is already known that its activation and signaling differ from other profibrotic cytokines such as *TGFB1* [47].

**Figure 4:**
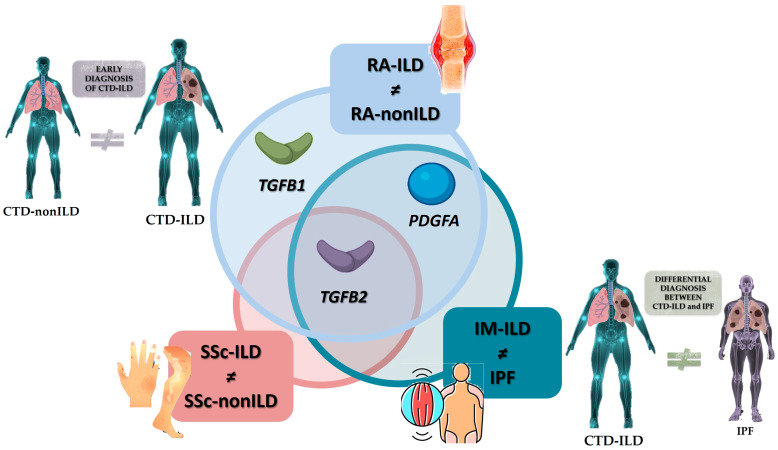
*PDGFA* and *TGFB* constitute promising biomarkers for the early and accurate diagnosis of CTD-ILD. CTD: connective tissue disease; ILD: interstitial lung disease; RA: rheumatoid arthritis; *TGFB*: transforming growth factor beta; *PDGFA*: platelet-derived growth factor subunit A; SSc: systemic sclerosis; IM: inflammatory myopathy; IPF: idiopathic pulmonary fibrosis.

Overall, our results point to a down-expression of *PDGFA* and *TGFB* in blood cells with the presence of lung fibrosis, as opposed to the overexpression reported at the tissue level. In this sense, several mechanisms could be involved in this pathological process observed in our CTD-ILD patients. We hypothesized that persistent fibrotic signaling might trigger systemic regulatory mechanisms that suppress the expression of genes in circulating cells, as part of a broader compensatory response. Alternatively, *PDGFA*/*TGFB*-expressing cells may preferentially migrate from the bloodstream into the fibrotic lung, leading to lower expression levels in the peripheral compartment. Moreover, tissue sequestration and local retention of profibrotic mediators may result in relative depletion from the blood. Finally, differences in epigenetic or regulatory landscapes between lung and blood compartments could contribute to divergent gene expression profiles. Therefore, we found circulating transcriptional changes as biomarkers of systemic disease modulation in CTD-ILD, rather than direct mirrors of lung tissue gene expression, reflecting indirect pathogenetic processes in our patients with CTD-ILD. In a clinical setting, our findings highlighted the great potential of *TGFB2* as a blood biomarker for the early identification of ILD in RA. Furthermore, we found that *PDGFA* and *TGFB1* had moderate capacity for the specific diagnosis of ILD in RA, *TGFB2* for identifying ILD in SSc, and *TGFB2* for discriminating IM-ILD from IPF. Additionally, *PDGFA* showed limited discriminatory capacity between IM-ILD and IPF. Finally, we found that *PDGFA*, *TGFB2*, and *TGFB3* can predict the prognosis of RA-ILD and SSc-ILD (Figure 4). These results point to these molecules are suitable candidates for further research, including longitudinal studies, to confirm their role and to determine whether they pre-date the development of ILD and could therefore be used as a predictive marker. Interestingly, these biological factors are measured using qPCR-based analysis, which is currently a well-established technique in diagnostic laboratories with high sensitivity and specificity, decreasing costs and increasing automation, making its integration into clinical workflows for the detection of gene expression biomarkers for CTD-ILD diagnosis increasingly feasible.

## 4. Materials and Methods

### 4.1. Patient Populations

A total of 289 patients from the Pneumology and Rheumatology departments of the Marqués de Valdecilla University Hospital (Santander, Cantabria, Spain) were recruited for the present study. On the one hand, the objective group comprised 93 patients with CTD-ILD, including patients with RA-ILD (*n* = 33), SSc-ILD (*n* = 31), and IM-ILD (*n* = 29). These patients met the established diagnostic criteria for each CTD. Specifically, they met the criteria established by the American College of Rheumatology (ACR) and European League Against Rheumatism (EULAR) for the classification and diagnosis of RA [48], SSc [49], and IM [50]. All patients with ILD also met the criteria for the classification and diagnosis of ILD as defined by the American Thoracic Society (ATS) and the European Respiratory Society (ERS) [51,52]. The presence of pulmonary fibrosis was confirmed in all patients by chest HRCT, and the degree of pulmonary functional involvement was assessed by PFTs. HRCT patterns in patients with ILD are stratified according to the criteria of the Fleischner Society and the American Society criteria as usual interstitial pneumonia (UIP) pattern, probable UIP pattern, indeterminate UIP pattern, and pattern with features more consistent with an alternative diagnosis (non-specific interstitial pneumonia (NSIP) or non-NSIP) [52,53]. Patients with CTD-ILD and a progressive fibrosing phenotype were diagnosed as such if they met one or more of the following ILD progression criteria over 24 months: relative decrease in predicted forced vital capacity (FVC) ≥ 10%; relative decrease in predicted FVC ≥5–<10% combined with increased extent of fibrosis on chest HRCT; relative decrease in predicted FVC ≥5–<10% combined with worsening respiratory symptoms; or a combination of worsening respiratory symptoms and increased extent of fibrosis on HRCT [54].

On the other hand, two control groups were included: patients with CTD without pulmonary complications (CTD-nonILD), and patients with ILD without an underlying CTD (IPF patients). In this regard, 48 patients with CTD-nonILD were recruited: 22 patients with RA, 18 with SSc, and 8 with IM. These patients met the aforementioned ACR/EULAR classification and diagnostic criteria [48,50,55] and were excluded from having ILD based on the presence of pulmonary fibrosis via chest HRCT and the degree of pulmonary functional involvement via PFTs. Furthermore, the project comprised 148 patients diagnosed with IPF recruited from the Pneumology Service of the HUMV. These patients met the who met the ATS/ETS criteria for ILD classification and diagnosis [51,52], with presence of NIU pattern in chest HRCT and the degree of pulmonary functional involvement assessed by PFTs.

As shown in Table 4, the following demographic and clinical information related to ILD and CTD was collected: sex, age at study, smoking habit, characteristic antibodies for the diagnosis of each CTD: rheumatoid factor (RF) and anti-cyclic citrullinated peptide antibodies (ACPA) for RA; antinuclear antibodies (ANA), anti-centromere antibodies (AAC) and anti-topoisomerase antibodies (ATA) (anti-Scl70) for SSc; and anti-histidyl tRNA synthetase antibodies (Anti-Jo-1); anti-threonyl tRNA synthetase antibodies (Anti-PL-7); anti-alanyl tRNA synthetase antibodiesanti-Jo1 (Anti-PL-12) for IM; ILD and CTD duration, degree of pulmonary involvement by PFTs, presence/absence of pulmonary fibrosis by HRCT, radiological pattern of fibrosis, presence/absence of progressive fibrosing phenotype, taking medication to control the disease (antifibrotic and/or immunosuppressive and/or vasodilator treatments).

All the experiments involving humans and human blood samples were carried out in accordance with the approved guidelines and regulations, according to the Declaration of Helsinki. All experimental protocols were approved by the Ethics Committee of Clinical Research of Cantabria, Spain (2016.092). All subjects gave written informed consent to participate in this study before their inclusion.

### 4.2. TGFB1, TGFB2, TGFB3, and PDGFA mRNA Expression Studies

The expression study was performed on RNA samples obtained from the peripheral blood of all patients included in this study using the commercial NucleoSpin^®^RNA Blood kit (Macherey-Nagel, Düren, NRW, Germany). The purity and concentration of total RNA were measured on the NanoDrop ND-1000 spectrophotometer (Thermo Scientific, Waltham, MA, USA), after which the RNA was stored at −80 °C until use.

The commercial RNA concentration kit GeneJET RNA Cleanup and Concentration Micro (Thermo Scientific, Waltham, MA, USA) was used to concentrate the RNA. Subsequently, the purified RNA was retrotranscribed to obtain cDNA using the iScript Advanced cDNA Synthesis kit for RT-qPCR (Bio-Rad, Hercules, CA, USA) using the PCR equipment “SureCycler 8800” (Agilent Technologies, Santa Clara, CA, USA). Gene expression was studied by qPCR using the “QuantStudio™ 7 Flex System” kit. Specific primers were designed for the interest genes (*TGFB1*, *TGFB2*, *TGFB3*, *PDGFA*) and housekeeping gene (*GAPDH*). A fluorescent intercalating agent (SYBR Green, Bio-Rad, Hercules, CA, USA), was used to amplify the DNA, producing a fluorescent signal that increased proportionally with the amount of PCR product. The average Ct value of each sample was obtained using the QuantStudio™ Real-Time PCR program (Applied Biosystem, Foster City, CA, USA). The relative expression of *TGFB1*, *TGFB2*, *TGFB3*, and *PDGFA* was calculated using the comparative Ct method, also known as the 2 delta-delta Ct (ΔΔCt) method, which represents the relative gene expression of the gene of interest in relation to *GAPDH* reference gene.

### 4.3. Statistical Analyses

The role of *TGFB* and *PDGF* expression in the early and accurate diagnosis and prognosis of CTD-ILD was evaluated. Firstly, their role in the early diagnosis of CTD-ILD was examined by comparing data from these patients with data from those with CTD-nonILD. Secondly, their role in accurately diagnosing CTD-ILD was evaluated by comparing data from these patients with that of patients with IPF. Finally, their role in the identification of CTD-ILD patients with a worse prognosis was analyzed by stratifying CTD-ILD patients according to the degree of pulmonary involvement determined by PFTs, the presence or absence of pulmonary fibrosis defined by HRCT, the radiological pattern of fibrosis and the presence or absence of a progressive fibrosing phenotype.

A comparison of gene expression values between two study groups was performed using analysis of variance (ANOVA), adjusting for the potential confounding factors of age, sex, and smoking status. The association of these values with continuous and categorical variables was analyzed by estimation of Pearson’s partial correlation coefficient (r) and linear regression, respectively, adjusting for the aforementioned potential confounding factors. The usefulness of these genes as potential biomarkers was tested by performing a receiver operating characteristic (ROC) curve analysis. The area under the curve (AUC) with a 95% confidence interval (CI) was calculated and the optimal cut-off value for discriminating between the two study groups was determined using the Youden index (the highest value obtained from the formula % sensitivity + % specificity − 100).

In all cases, a *p*-value of *p* ≤ 0.05 was considered statistically significant. All statistical analyses were performed using the STATA 12/SE statistical program (Stata Corp., College Station, TX, USA) and GraphPad Prism 5 software.

## 5. Conclusions

In conclusion, *PDGFA* and *TGFB* could be promising blood biomarkers of systemic disease modulation in CTD-ILD. As part of a markers broader panel and when combined with other clinical, imaging, or functional parameters, they may have potential clinical value in the early and accurate diagnosis of CTD-ILD, helping to overcome the challenges posed by this disease in clinical practice.

## Figures and Tables

**Table 1:** Relationship of *PDGFA*, *TGFB1*, *TGFB2*, and *TGFB3* gene expression with clinical characteristics of RA-ILD patients.

	*PDGFA*		*TGFB1*		*TGFB2*		*TGFB3*	
Variable	r	*p*	r	*p*	r	*p*	r	*p*
RA duration	0.23	0.24	0.37	0.05	−0.07	0.74	0.06	0.78
ILD duration	−0.06	0.77	0.17	0.38	−0.15	0.42	−0.09	0.63
FVC (% predicted)	−0.21	0.28	−0.22	0.26	−0.16	0.40	0.22	0.25
FEV1 (% predicted)	−0.19	0.31	−0.27	0.16	−0.21	0.29	0.35	0.07
DLCO (% predicted)	0.04	0.88	−0.36	0.13	0.17	0.48	−0.06	0.82
Category	MEAN ± SD	*p*	MEAN ± SD	*p*	MEAN ± SD	*p*	MEAN ± SD	*p*
RF^−^	0.0162 ± 0.0067	0.43	0.3314 ± 0.0213	0.16	0.0011 ± 0.0006	0.97	0.0008 ± 0.0002	0.65
RF^+^	0.0130 ± 0.0060		0.2531 ± 0.0833		0.0012 ± 0.0018		0.0077 ± 0.0353	
ACPA^−^	0.0131 ± 0.0073	0.89	0.2560 ± 0.0772	0.95	0.0032 ± 0.0048	**0.01**	0.0018 ± 0.0026	0.97
ACPA^+^	0.0134 ± 0.0060		0.2611 ± 0.0847		0.0009 ± 0.0005		0.0078 ± 0.0360	
UIP pattern, probable UIP pattern or Indeterminate for UIP pattern	0.0120 ± 0.0058	**0.04**	0.2499 ± 0.0794	0.17	0.0013 ± 0.0020	0.72	0.0010 ± 0.0011	0.52
NSIP pattern or Non-NSIP pattern	0.0161 ± 0.0053		0.2939 ± 0.0867		0.0010 ± 0.0052		0.0196 ± 0.0592	
Progressive pulmonary fibrosis^−^	0.0137 ± 0.0057	0.33	0.2739 ± 0.0778	0.09	0.0012 ± 0.0018	0.80	0.0077 ± 0.0353	0.92
Progressive pulmonary fibrosis^+^	0.0109 ± 0.0073		0.2032 ± 0.0938		0.0010 ± 0.0007		0.0008 ± 0.0004	
Antifibrotic treatment^−^	0.0134 ± 0.0059	0.37	0.2650 ± 0.0836	0.48	0.0012 ± 0.0017	0.57	0.0070 ± 0.0359	0.95
Antifibrotic treatment^+^	0.0080 ± 0.0000		0.2052 ± 0.0000		0.0019 ± 0.0000		0.0014 ± 0.0000	
sDMARDs^−^	0.0118 ± 0.0040	0.31	0.2595 ± 0.0830	0.90	0.0009 ± 0.0005	0.29	0.0008 ± 0.0004	0.40
sDMARDs^+^	0.0140 ± 0.0068		0.2649 ± 0.0876		0.0015 ± 0.0023		0.0121 ± 0.0453	
bDMARDs^−^	0.0125 ± 0.0048	0.58	0.2425 ± 0.0730	0.08	0.0009 ± 0.0005	0.28	0.0106 ± 0.0429	0.52
bDMARDs^+^	0.0136 ± 0.0071		0.2954 ± 0.0938		0.0017 ± 0.0028		0.0012 ± 0.0015	

*PDGFA*: platelet-derived growth factor subunit A; *TGFB*: transforming growth factor beta; RA: rheumatoid arthritis; ILD: interstitial lung disease; FVC: forced vital capacity; FEV1: forced expiratory volume 1; DLCO: diffusing capacity for carbon monoxide; RF: rheumatoid factor; ACPA: anti-cyclic citrullinated peptide antibodies; UIP: usual interstitial pneumonia; NSIP: non-specific interstitial pneumonia; sDMARDs: synthetic disease-modifying antirheumatic drugs; bDMARDs: biologic disease-modifying antirheumatic drugs; SD: standard deviation. Significant results are indicated in **bold**.

**Table 2:** Relationship of *PDGFA*, *TGFB1*, *TGFB2*, and *TGFB3* gene expression with clinical characteristics of SSc-ILD patients.

	*PDGFA*		*TGFB1*		*TGFB2*		*TGFB3*	
Variable	r	*p*	r	*p*	r	*p*	r	*p*
SSc duration	0.04	0.84	−0.15	0.45	−0.13	0.53	−0.02	0.91
ILD duration	−0.12	0.54	−0.15	0.46	−0.22	0.27	−0.06	0.78
FVC (% predicted)	0.24	0.22	0.07	0.74	0.35	0.08	−0.21	0.33
FEV1 (% predicted)	0.30	0.13	0.16	0.42	0.40	**0.04**	−0.18	0.39
DLCO (% predicted)	0.09	0.70	−0.18	0.44	0.22	0.38	−0.21	0.40
Category	MEAN ± SD	*p*	MEAN ± SD	*p*	MEAN ± SD	*p*	MEAN ± SD	*p*
ANAs^−^	0.0044 ± 0.0000	0.10	0.3220 ± 0.0000	0.71	0.0001 ± 0.0000	0.30	0.0004 ± 0.0000	0.63
ANA^+^	0.0155 ± 0.0652		0.2604 ± 0.6789		0.0013 ± 0.0015		0.0028 ± 0.0096	
ACA^−^	0.0154 ± 0.0067	0.46	0.2624 ± 0.0688	0.94	0.0013 ± 0.0015	0.66	0.0028 ± 0.0096	0.97
ACA^+^	0.0070 ± 0.0000		0.2639 ± 0.0000		0.0007 ± 0.0000		0.0007 ± 0.0000	
ATA (anti-Scl-70)^−^	0.0126 ± 0.0036	0.13	0.2609 ± 0.0335	0.72	0.0012 ± 0.0018	0.92	0.0011 ±0.0005	0.29
ATA (anti-Scl-70)^+^	0.0172 ± 0.0076		0.2673 ± 0.0839		0.0013 ± 0.0015		0.0040 ± 0.0125	
UIP pattern, probable UIP pattern or Indeterminate for UIP pattern	0.0137 ± 0.0077	0.88	0.2580 ± 0.0859	0.70	0.0005 ± 0.0002	0.08	0.0069 ± 0.0177	**0.02**
NSIP pattern or Non-NSIP pattern	0.0153 ± 0.0067		0.2618 ± 0.0638		0.0016 ± 0.0017		0.0010 ± 0.0007	
Progressive pulmonary fibrosis^−^	0.0153 ± 0.0646	0.76	0.2613 ± 0.0607	0.48	0.0013 ± 0.0014	0.86	0.0009 ± 0.0006	**0.02**
Progressive pulmonary fibrosis^+^	0.0135 ± 0.0078		0.2569 ± 0.0896		0.0013 ± 0.0019		0.0073 ± 0.0176	
Antifibrotic treatment^−^	0.0149 ± 0.0067	0.60	0.2587 ± 0.0687	0.86	0.0013 ± 0.0015	0.46	0.0028 ± 0.0096	0.89
Antifibrotic treatment^+^	0.0146 ± 0.0095		0.2813 ± 0.0670		0.0006 ± 0.0002		0.0007 ± 0.0005	
sDMARDs^−^	0.0152 ± 0.0040	0.95	0.2505 ± 0.0676	0.30	0.0018 ± 0.0021	0.56	0.0011 ± 0.0005	0.77
sDMARDs^+^	0.0151 ± 0.0076		0.2663 ± 0.0738		0.0012 ± 0.0014		0.0033 ± 0.0112	
bDMARDs^−^	0.0159 ± 0.0069	0.88	0.2459 ± 0.0619	0.14	0.0017 ± 0.0020	0.40	0.0047 ± 0.0138	0.65
bDMARDs^+^	0.0156 ± 0.0076		0.2971 ± 0.0801		0.0011 ± 0.0009		0.0010 ± 0.0009	
Vasodilators treatment^−^	0.0156 ± 0.0678	0.78	0.2956 ± 0.0670	0.46	0.0012 ± 0.0009	0.42	0.0010 ± 0.0008	0.91
Vasodilators treatment^+^	0.0159 ± 0.0074		0.2527 ± 0.0732		0.0016 ± 0.0019		0.0042 ± 0.1288	

*PDGFA*: platelet-derived growth factor subunit A; *TGFB*: transforming growth factor beta; SSc: systemic sclerosis; ILD: interstitial lung disease; FVC: forced vital capacity; FEV1: forced expiratory volume 1; DLCO: diffusing capacity for carbon monoxide; ANA: antinuclear antibody; ACA: anti-centromere antibodies; ATA: anti-topoisomerase I antibodies; UIP: usual interstitial pneumonia; NSIP: non-specific interstitial pneumonia; sDMARDs: synthetic disease-modifying antirheumatic drugs; bDMARDs: biologic disease-modifying antirheumatic drugs; SD: standard deviation. Significant results are indicated in **bold**.

**Table 3:** Relationship of *PDGFA*, *TGFB1*, *TGFB2*, and *TGFB3* gene expression with clinical characteristics of IM-ILD patients.

	*PDGFA*		*TGFB1*		*TGFB2*		*TGFB3*	
Variable	r	*p*	r	*p*	r	*p*	r	*p*
IM duration	−0.20	0.32	0.02	0.93	0.01	0.96	0.17	0.43
ILD duration	0.14	0.53	0.02	0.92	−0.16	0.48	0.14	0.53
FVC (% predicted)	0.05	0.80	−0.03	0.88	−0.16	0.48	0.01	0.96
FEV1 (% predicted)	0.08	0.73	0.01	0.97	−0.07	0.74	−0.04	0.85
DLCO (% predicted)	−0.09	0.75	−0.11	0.70	−0.08	0.80	0.43	0.14
Category	MEAN ± SD	*p*	MEAN ± SD	*p*	MEAN ± SD	*p*	MEAN ± SD	*p*
Anti-Jo1^−^	0.0216 ± 0.0201	0.90	0.3334 ± 0.1490	0.81	0.0013 ± 0.0009	0.38	0.0073 ± 0.0284	0.59
Anti-Jo1^+^	0.0236 ± 0.0234		0.3513 ± 0.1891		0.0009 ± 0.0007		0.0006 ± 0.0006	
Anti-PL7^−^	0.0226 ± 0.0198	0.79	0.3507 ± 0.1601	0.40	0.0012 ± 0.0008	0.50	0.0072 ± 0.0284	0.61
Anti-PL7^+^	0.0195 ± 0.0236		0.2822 ± 0.1169		0.0016 ± 0.0011		0.0010 ± 0.0005	
Anti-PL12^−^	0.0222 ± 0.0212	0.82	0.3329 ± 0.1467	0.72	0.0012 ± 0.0009	0.83	0.0067 ± 0.0272	0.72
Anti-PL12^+^	0.0193 ± 0.0094		0.3679 ± 0.2380		0.0011 ± 0.0006		0.0010 ± 0.0007	
UIP pattern, probable UIP pattern or Indeterminate for UIP pattern	0.0251 ± 0.0199	0.73	0.3454 ± 0.1684	0.89	0.0013 ± 0.0009	0.60	0.0011 ± 0.0007	0.11
NSIP pattern or Non-NSIP pattern	0.0201 ± 0.0249		0.3279 ± 0.1572		0.0011 ± 0.0010		0.0007 ± 0.0005	
Progressive pulmonary fibrosis^−^	0.0213 ± 0.0204	0.62	0.3346 ± 0.1444	0.81	0.0012 ± 0.0010	0.68	0.0070 ± 0.0285	0.57
Progressive pulmonary fibrosis^+^	0.0287 ± 0.0312		0.3197 ± 0.3097		0.0014 ± 0.0008		0.0008 ± 0.0008	
Antifibrotic treatment^−^	0.0225 ± 0.0216	0.71	0.3498 ± 0.1542	0.11	0.0012 ± 0.0009	0.96	0.0067 ± 0.0278	0.99
Antifibrotic treatment^+^	0.0107 ± 0.0030		0.1421 ± 0.0526		0.0011 ± 0.0009		0.0004 ± 0.0001	
sDMARDs^−^	0.0282 ± 0.0272	0.60	0.3761 ± 0.1588	0.29	0.0016 ± 0.0011	0.11	0.0010 ± 0.0006	0.09
sDMARDs^+^	0.0173 ± 0.0107		0.2997 ± 0.1489		0.0009 ± 0.0007		0.0101 ± 0.0344	
bDMARDs^−^	0.0261 ± 0.0223	0.21	0.3537 ± 0.1721	0.42	0.0012 ± 0.0009	0.98	0.0075 ± 0.0291	0.84
bDMARDs^+^	0.0111 ± 0.0060		0.2796 ± 0.0732		0.0012 ± 0.0010		0.0009 ± 0.0006	

*PDGFA*: platelet-derived growth factor subunit A; *TGFB*: transforming growth factor beta; IM: inflammatory myopathy; ILD: interstitial lung disease; FVC: forced vital capacity; FEV1: forced expiratory volume 1; DLCO: diffusing capacity for carbon monoxide; Anti-Jo1: Anti-histidyl tRNA synthetase antibodies; Anti-PL7: Anti-threonyl tRNA synthetase antibodies; Anti-PL12: Anti-alanyl tRNA synthetase antibodies; UIP: usual interstitial pneumonia; NSIP: non-specific interstitial pneumonia; sDMARDs: synthetic disease-modifying antirheumatic drugs; bDMARDs: biologic disease-modifying antirheumatic drugs; SD: standard deviation.

**Table 4:** Demographic and clinical characteristics of all the patients of the study.

	Study Objective Groups			Comparative Groups			
	RA-ILD *n* = 33	SSc-ILD *n* = 31	IM-ILD *n* = 29	RA-nonILD *n* = 22	SSc-nonILD *n* = 18	IM-nonILD *n* = 8	IPF *n* = 148
Sex (women), n (%)	13 (39.39)	19 (61.29)	18 (62.07)	14 (63.64)	16 (88.89)	6 (75.00)	123 (83.11)
Age at study (years), mean ± SD	70.73 ± 7.1	63.06 ± 10.2	62.45 ± 19.0	64.68 ± 11.29	57.94 ± 13.76	63.86 ± 19.17	70.60 ± 7.12
Smoking ever, n (%)	26 (78.79)	16 (51.61)	21 (72.41)	13 (59.09)	10 (55.56)	3 (37.5)	130 (87.84)
Antibody status							
RF^+^, n (%)	29 (87.87)	-	-	12 (54.55)	-	-	-
ACPA^+^, n (%)	28 (84.84)	-	-	14 (63.64)	-	-	-
ANA^+^, n (%)	-	29 (93.55)	-	-	16 (88.89)	-	-
ACA^+^, n (%)	-	1 (3.23)	-	-	8 (44.44)	-	-
ATA (anti-Scl70)^+^, n (%)	-	17 (54.84)	-	-	3 (16.67)	-	-
Anti-Jo1^+^, n (%)	-	-	5 (17.24)	-	-	0 (0.00)	-
Anti-PL7^+^, n (%)	-	-	6 (20.69)	-	-	1 (12.5)	-
Anti-PL12^+^, n (%)	-	-	3 (10.34)	-	-	0 (0.00)	-
CTD duration (years), mean ± SD	6.72 ± 7.13	7.01 ± 7.06	7.96 ± 21.29	9.70 ± 9.65	9.94 ± 7.92	5.27 ± 1.58	-
ILD duration (years), mean ± SD	3.88 ± 4.28	5.64 ± 6.95	3.88 ± 4.28	-	-	-	3.43 ± 3.49
Pulmonary function tests							
FVC (% predicted), mean ± SD	85.46 ± 26.60	78.75 ± 24.16	79.58 ± 24.77	103.90 ± 17.51	107.20 ± 16.12	113.00 ± 0.00	75.73 ± 18.94
FEV1 (% predicted), mean ± SD	83.82 ± 24.52	77.11 ± 22.99	81.21 ± 25.57	101.10 ± 22.72	102.50 ±18.14	119.00 ± 0.00	77.57 ± 19.39
DLCO (% predicted), mean ± SD	44.34 ± 19.50	39.60 ± 17.71	52.18 ± 16.21	77.49 ± 17.85	70.68 ± 15.46	101.0 ± 0.00	36.47 ± 16.11
HRCT							
Pulmonary involvement in HRCT, n (%)	33 (100.0)	31 (100.0)	29 (100.0)	0 (0.00)	0 (0.00)	0 (0.00)	148 (100.00)
UIP pattern, n (%)	17 (51.52)	8 (25.80)	7 (24.14)	-	-	-	148 (100.00)
Probable UIP pattern, n (%)	5 (15.15)	1 (3.23)	6 (20.69)	-	-	-	0 (0.00)
Indeterminate for UIP pattern, n (%)	1 (3.03)	0 (0.00)	0 (0.00)	-	-	-	0 (0.00)
NSIP pattern, n (%)	9 (27.27)	20 (64.52)	9 (31.03)	-	-	-	0 (0.00)
Non-NSIP pattern, n (%)	1 (3.03)	2 (6.45)	2 (6.90)	-	-	-	0 (0.00)
Progressive pulmonary fibrosis, n (%)	5 (15.15)	8 (25.81)	3 (10.34)	-	-	-	148 (100.00)
Treatments							
Antifibrotics, n (%)	1 (3.03)	2 (6.45)	2 (6.90)	-	-	-	98 (66.21)
sDMARDs, n (%)	17 (52.52)	22 (70.97)	15 (51.72)	19 (86.36)	11 (61.11)	6 (75.00)	-
bDMARDs, n (%)	12 (36.36)	10 (32.26)	7 (24.14)	4 (18.18)	2 (36.00)	3 (37.5)	-
Vasodilatators, n (%)	-	17 (54.84)	-	-	13 (72.22)	-	-

RA: rheumatoid arthritis; ILD: interstitial lung disease; RF: rheumatoid factor; ACPA: anti-cyclic citrullinated peptide antibodies; ANA: antinuclear antibodies; ACA: anti-centromere antibodies; ATA (anti-scl-70): anti-topoisomerase I antibodies; Anti-Jo-1: anti-histidyl tRNA synthetase antibodies; Anti-PL-7: anti-threonyl tRNA synthetase antibodies; Anti-PL-12: anti-alanyl tRNA synthetase antibodies; CTD: connective tissue diseases; ILD: interstitial lung disease; FVC: forced vital capacity; FEV1: forced expiratory volume in the first second; DLCO: diffusing capacity for carbon monoxide; HRCT: high-resolution computed tomography; UIP: usual interstitial pneumonia; NSIP: non-specific interstitial pneumonia; sDMARDs: synthetic disease-modifying antirheumatic drugs; bDMARDs: biologic disease-modifying anti-rheumatic drugs; SSc: systemic sclerosis; IM: inflammatory myopathies; IPF: idiopathic pulmonary fibrosis.

## Data Availability

All data generated or analyzed during this study are included in this published article [and its Supplementary Information Files].

## Supplementary Materials

The following supporting information can be downloaded at: https://www.mdpi.com/article/10.3390/ijms262110722/s1.

## Abbreviations

ACA	anti-centromere antibodies.
ACPA	anti-cyclic citrullinated peptide antibodies.
ACR	American College of Rheumatology.
ANA	anti-nuclear antibodies.
Anti-Jo-1	anti-histidyl tRNA synthetase antibodies.
Anti-PL-7	anti-threonyl tRNA synthetase antibodies.
Anti-PL-12	anti-alanyl tRNA synthetase antibodies.
ATA	anti-topoisomerase I antibodies.
ATS	American Thoracic Society.
ANOVA	analysis of variance.
AUC	area under the curve.
bDMARDs	biologic disease-modifying anti-rheumatic drugs.
CI	confident interval.
csDMARDs	conventional synthetic disease-modifying anti-rheumatic drugs.
CTDs	connective tissue diseases.
ERS	European Respiratory Society.
EULAR	European League Against Rheumatism.
FEV1	forced expiratory volume in one second.
FVC	forced vital capacity.
HRCT	high-resolution computed tomography.
ILD	interstitial lung disease.
IM	inflammatory myopathies.
IPF	idiopathic pulmonary fibrosis.
PDGF	platelet-derived growth factor.
PFTs	pulmonary function tests.
RA	rheumatoid arthritis.
ROC	receiver operating characteristic.
SD	standard deviation.
SSc	systemic sclerosis.
TGF-β	transforming growth factor-β.
UIP	usual interstitial pneumonia.:
