# Supplementary material for: A New Approach for Achieving Earlier and More Accurate Diagnosis of Connective Tissue Disease-Related Interstitial Lung Disease: TGFB and PDGFA as Novel Promising Biomarkers
Source: Int J Mol Sci. 2025 Nov 4;26(21):10722. doi: 10.3390/ijms262110722 (PMC12609778; doi:10.3390/ijms262110722)
Supplement: Supplementary file 1 [file ijms-26-10722-s001.zip › ijms-3933067-supplementary.pdf]

Table S1. ROC curves analysis for the discrimination of RA-ILD from RA-nonILD, SSc-ILD from SSc-nonILD, and IM-ILD from IPF.

	AUC (95% CI)	p-value	Optimal cut-off value	Sensitivity (%)	Specificity (%)
**RA-ILD *vs* RA-nonILD**					
PDGFA	0.7345 (0.5987–0.8703)	**0.0039520**	< 0.01153	54.55	90.48
TGFB1	0.7300 (0.5831–0.8770)	**0.0041340**	< 0.31850	75.76	72.73
TGFB2	0.8021 (0.6800–0.9242)	**0.0002253**	< 0.00141	84.38	66.67
**SSc-ILD *vs* SSc-nonILD**					
TGFB1	0.6452 (0.4753 - 0.8150)	0.0930500	-	-	-
TGFB2	0.7518 (0.6028 - 0.9009)	**0.0040240**	< 0.00180	87.50	58.82
**IM-ILD *vs* IPF**					
PDGFA	0.6179 (0.4981 - 0.7377)	**0.0369200**	> 0.01660	46.88	81.51
TGFB2	0.7064 (0.5959 - 0.8170)	**0.0002847**	> 0.00155	43.75	94.89

ROC: receiver operating characteristic; RA: rheumatoid arthritis; ILD: interstitial lung diseases; SSc: systemic sclerosis; IM; inflammatory myophaties; IPF: idiopathic pulmonary fibrosis; AUC: area under the curve; CI: confidence interval; PDGFA: platelet-derived growth factor subunit A; AUC: area under the curve; TGFB: transforming growth factor beta. Significant results are highlighted in **bold**.
